# Optimizing renal biopsy: efficacy and safety of the ultrasound-guided tangential approach

**DOI:** 10.1590/0100-3984.2024.0132-en

**Published:** 2025-06-16

**Authors:** Rogério Augusto Pinto-Silva, Marcell de Barros Duarte Pereira, Milton Domingos Panzi Neto, Otton Lourenço de Lima Reis, Raquel Sadala Mendes, Gessilane Martins da Silva, David Campos Wanderley, Stanley de Almeida Araújo

**Affiliations:** 1 Clínica CEU Diagnósticos, Belo Horizonte, MG, Brazil; 2 Instituto de Nefropatologia, Belo Horizonte, MG, Brazil

**Keywords:** Image-guided biopsy, Kidney diseases, Ambulatory care, Ultrasonography, interventional., Biópsia guiada por imagem, Doenças renais, Pacientes ambulatoriais, Ultrassonografia intervencionista.

## Abstract

**Objective:**

To present the results of a series of outpatient renal biopsies performed with a tangential approach, as well as to conduct an analysis focusing on patient safety and the frequency with which sufficient material was obtained.

**Materials and Methods:**

This retrospective observational study examined the pathology results and evolution of 244 patients referred for ultrasound-guided renal biopsy at a single center. In each biopsy, the needle was advanced in the cortex just below the renal capsule. The pathologist examined the fragments, counting the viable glomeruli obtained; additional punctures were performed if necessary, as long as Doppler ultrasound showed no bleeding. The patients remained at rest at the clinic, being discharged after a follow-up ultrasound evaluation and contacted one week later to investigate late adverse events.

**Results:**

Ten patients were excluded from the analysis, leaving a sample of 234 patients. The material obtained for diagnosis was considered sufficient in 95.73% of the procedures, partially adequate in 3.42%, and not very representative in 0.85%. Two patients (0.85%) had bleeding greater than 50 cm^3^ and were referred to the hospital emergency department. Both of those patients had a favorable evolution: one required only a period at rest, and the other required a blood transfusion, being discharged 48 h after the procedure.

**Conclusion:**

The tangential approach to renal biopsy, with its high rates of safety and efficacy, representing a reliable diagnostic tool for renal and systemic diseases, should be the method of choice for obtaining adequate pathological specimens.

## Abstract (pt)

**Objetivo:**

Apresentamos uma série de biópsias renais ambulatoriais utilizando o método tangencial, analisando a frequência de material suficiente e a segurança para o paciente.

**Materiais e Métodos:**

Revimos os laudos anatomopatológicos e a evolução de 244 pacientes encaminhados para biópsia renal ecoguiada em um único centro, disparando a agulha logo abaixo da cápsula renal tangenciando o córtex. Os fragmentos foram examinados pelo patologista, que contou os glomérulos viáveis obtidos. Se necessárias, novas punções eram realizadas, desde que o paciente não apresentasse sangramento ao Doppler. O paciente permanecia em repouso na clínica e era liberado após revisão. Após uma semana, o paciente era contatado para investigação de eventos adversos tardios.

**Resultados:**

Dez pacientes foram excluídos da série, restando 234 para análise. Material suficiente para diagnóstico foi obtido em 95,73% dos casos, parcialmente adequado em 3,42% e pouco representativo em 0,85%. Dois pacientes (0,85%) apresentaram sangramento maior que 50 cm^3^ e foram encaminhados para serviço de pronto-atendimento hospitalar, e ambos tiveram evolução favorável: um permaneceu apenas em repouso e o outro necessitou de transfusão sanguínea, tendo alta após 48 horas.

**Conclusão:**

A abordagem tangencial para biópsia renal, com suas altas taxas de segurança e eficácia, representa uma ferramenta diagnóstica confiável para doenças renais e sistêmicas e deve ser o método de escolha para obtenção de espécimes patológicos adequados.

## INTRODUCTION

Various kidney diseases, especially nephrotic syndrome, nephritic syndrome, and worsening of renal function in native or transplanted kidneys, require histopathological evaluation of the parenchyma (renal biopsy) for etiological diagnosis and appropriate treatment. Renal biopsy is also quite valuable for evaluating systemic diseases with renal involvement, such as diabetes mellitus, collagenosis, and amyloidosis. It has become an indispensable tool not only for diagnosis but also for defining the treatment and prognosis^(1)^. One objective of renal biopsy in renal parenchymal diseases (glomerulonephritis, renal dysfunction, etc.), as well as for the evaluation of renal grafts, is to obtain at least 20 glomeruli for study under light microscopy with immunofluorescence and at least one glomerulus for study under electron microscopy, the latter only as necessary.

The use of renal biopsy in conjunction with imaging methods has allowed the acquisition of better samples with greater safety. Ultrasound stands out because it enables the procedure to be visualized in real time, without exposing the patient to radiation or potentially nephrotoxic contrast agents, and can be performed on an outpatient basis^**(^2^)**^. In addition to anatomical imaging by B-mode, color Doppler can locate blood vessels in real time, avoiding inadvertent punctures and demonstrating active bleeding after the needle has been withdrawn.

The development of automatic and semi-automatic devices using tru-cut needles has improved the quality of the material obtained in renal biopsy and increased the safety of the procedure. In those devices, the needle advance is standardized (typically 1.5-2.2 cm), allowing the physician to calculate the needle trajectory, thus avoiding puncturing arteries, which are visualized with color Doppler^**(^3^)**^.

We present the results obtained with ultrasound-guided renal biopsies performed at a single (private) center in Brazil by a team of radiologists and pathologists. The pathologists evaluated the fresh fragment under light microscopy, counting the glomeruli and guiding the sonographer regarding the need for additional punctures. This immediate evaluation allowed us to improve the technique, obtain more glomeruli per pass, virtually eliminate inconclusive reports due to insufficient material, and provide greater patient safety, given the very low rate of adverse events. A review of the literature revealed that the technique we were using is called the tangential or cortical tangential approach, and it has been described in a relatively small number of articles, which motivated us to conduct this study^(4-8)^. This article will not address biopsies of focal renal lesions.

The purpose of the study was to report the technique of ultrasound-guided percutaneous renal cortical biopsy with a tangential approach and the results achieved, especially regarding the quality of the material obtained (based on the number of glomeruli), the proportion of sufficient samples, and the frequency of adverse events.

## MATERIALS AND METHODS

This was a retrospective observational study of all consecutive renal biopsies performed during 2023 at a private clinic in the city of Belo Horizonte, Brazil. The patients had been referred by nephrologists in the state of Minas Gerais, most in the metropolitan region of Belo Horizonte. To schedule a consultation, the patient presented a physician referral with justification and the results of laboratory tests performed in the last 40 days: determination of the coagulation profile; serum urea and creatinine; routine urine testing; and urine microscopy or culture (to identify bacteriuria). Patients were considered candidates for biopsy if they had an international normalized ratio (INR) ≤ 1.5, an activated partial thromboplastin time (APTT) ≤ 10 s above the control, a platelet count ≥ 50,000/mm^3^, no gram-negative bacilli on microscopy or urine culture, or < 100,000 colonies of gram-negative bacilli on urine culture^**(^3^)**^. Patients were accepted even if they were already undergoing treatment for a urinary tract infection. Patients with coagulation disorders were also accepted if the alteration had been corrected by a hematologist. Patients using anticoagulants or antiplatelet drugs were required to provide a form attesting that those medications had been discontinued, signed by the requesting physician or hematologist, as well as verbal confirmation of the discontinuation of their use. A renal imaging examination was ordered, and patients in whom there was evidence of significant bilateral renal atrophy were excluded.

Before the biopsy, patients were provided with a description of the procedure, as well as of any adverse events, and any questions they had were answered, after which they gave written informed consent. Any patients with a diastolic blood pressure above 110 mmHg were sent to an adjoining room, where they rested in a quiet environment. If their diastolic pressure dropped to 110 mmHg or below, the biopsy was performed; otherwise, the patient was referred to the attending physician, who requested an adjustment of the dose of the antihypertensive medication.

All biopsies were performed in the morning in a room equipped with one of two ultrasound systems-a Logiq E9 (GE HealthCare, Milwaukee, WI, USA) or an Aplio A (Canon Medical Systems, Otawara, Japan)-both with the capability to perform conventional and microvascular color Doppler, as well as microbubble contrast imaging. The biopsies were conducted by a team of two physicians: one to the left of the examination table, operating the ultrasound system, and the other to the right, in front of the ultrasound monitor, manipulating the biopsy needle.

**Figure 1:**
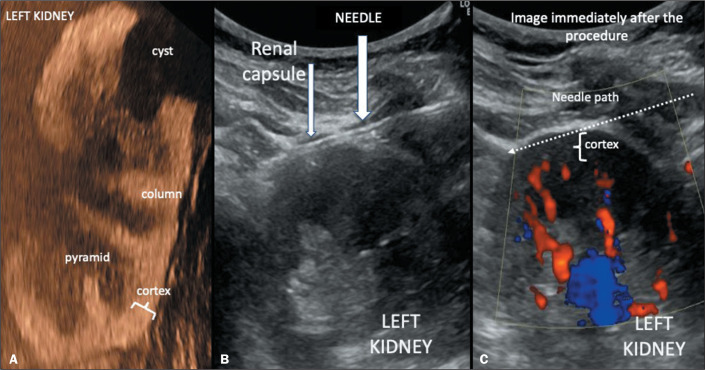
A: Arterial-phase ultrasound image with microbubble contrast showing hyperenhancement of the cortex and hypoenhancement of the renal pyramids, which are poorly defined in the normal kidney. The cortex above the pyramids is only 5 mm thick. B: B-mode ultrasound image of a cortical tangential biopsy of the left kidney: the needle passes just below the renal capsule, collecting material only from the renal cortex, avoiding the interlobar arteries, which are visible on color Doppler (C) mapping (scale limit: 11 cm/s) and appear in red in the image on the right, taken immediately after the needle was removed, in which no blood reflux is observed along the needle path (dotted arrow). In this image, it is clear that the subcapsular cortical tangential path does not reach the interlobar or arcuate arteries. Care should be taken to avoid a puncture in the region close to cysts, because they can deform the cortex and change the position of the arteries.

On the day of the examination, an ultrasound evaluation of the kidneys was performed. Patients were rejected if their kidneys had an atrophic appearance with a below-normal volume (typically less than 70-80 cm^3^ for each kidney, depending on the biotype). When both kidneys had a normal appearance, we gave preference to biopsy of the middle third or lower-middle third of the left kidney. If there was evidence of chronic disease that was more advanced in the left kidney than in the right kidney, or if the left kidney showed a below-normal volume or poor visualization on ultrasound, the right kidney was biopsied. For each biopsy, the patient was placed in the prone position or, in the case of obese patients, in the right lateral position, which allows an adequate window for biopsy of the left kidney. None of the patients were submitted to sedation or general anesthesia. After antisepsis with chlorhexidine and 2% surfactants, ultrasound-guided anesthesia was performed with 19 mL of 1% lidocaine without vasoconstrictor, augmented with 1 mL of 8.4% sodium bicarbonate, from the point of entry of the needle into the skin to the chosen site next to the renal capsule, typically in the middle third or lower-middle third of the kidney. The puncture was then performed with a tru-cut needle (16-gauge × 16 cm or 16-gauge × 20 cm) mounted on an automatic trigger device (Bard Magnum, Covington, GA, USA), with 22 mm of programmed advancement. The penetration of the biopsy needle was monitored by real-time ultrasound, allowing visualization of the kidney in cross-section, thus avoiding areas of scarring or renal cysts. After the needle tip had pierced the capsule, the needle was positioned so that advancement occurred in the region immediately below the capsule (Figure 1); that is, a tangential approach was taken. After the needle was triggered and removed, the puncture site was examined by color Doppler ultrasound, with a scale of 9-12 cm/s, to identify signs of active bleeding^**(^9^)**^. If no bleeding was detected, another puncture was performed.

**Figure 2:**
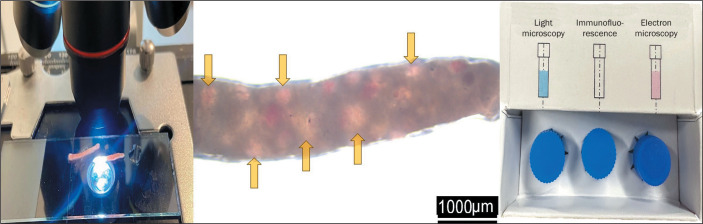
Microscopic examination of a fresh renal specimen obtained by using the tangential approach with a 16-gauge tru-cut needle. In the center, the appearance of the fragment under light microscopy, with arrows indicating some of the 14 glomeruli in the sample. The pathologist divides the fragment and places it in the vials supplied with the kit (panel on the right), with specific media for conventional histopathologic staining for (left to right) light microscopy, immunofluorescence, and electron microscopy.

After two punctures, the fragments obtained were examined under light microscopy in the biopsy room by the pathologist, who counted the nonsclerotic glomeruli (which appear as reddish spots because they contain red blood cells, whereas sclerotic glomeruli do not). The pathologist then informed the radiologists as to whether the material was suitable for the pathology examinations, including conventional histopathology, immunofluorescence, and electron microscopy, which, collectively, require the analysis of at least 20 glomeruli (Figure 2). If necessary, additional punctures were performed until that number was attained or Doppler revealed bleeding along the needle path. The pathologist sectioned the specimens and stored them in flasks with specific preservation media.

After the procedure, the patient was taken to the recovery room, where they remained in the right or left lateral position on a cushion, depending on which native kidney was punctured, or in the prone position in the case of a transplanted kidney, for 1-5 h (until there was no longer any bleeding), and was reassessed by ultrasound at the end of that period. Patients without complaints and without evidence of significant bleeding on ultrasound and Doppler were then discharged, taking with them a report of the procedure and the request for a pathology analysis at the Nephropathology Institute, also located in Belo Horizonte. The pathology analysis request form included patient sex, age, coagulation profile, and renal function, as well as the indication for the biopsy, kidney biopsied, number of fragments obtained, number of the vial containing the material, and possible adverse events.

If there was active bleeding, as seen on color Doppler, for more than five minutes, formation of a perinephric collection of 50-100 cm^3^, or a significant reduction in blood pressure in relation to the baseline value, venous access was obtained, fluid resuscitation was initiated, and the patient was transferred to the hospital emergency room. For patients with bleeding who had a serum level of urea > 100 mg/dL (16.65 mmol/L), a serum level of creatinine > 1.5 mg/dL (0.133 mmol/L), or both, desmopressin was administered intravenously at a dose of 0.3 µg/kg of body weight, diluted in 50-100 mL of saline solution. The patient or a family member was contacted one week after the biopsy, to track the progress of the patient and identify any late adverse events, which were classified as mild, moderate, or severe according to the time and resources required for their correction^**(^10^)**^.

## RESULTS

During the study period (January to December of 2023), renal biopsy was performed in 244 patients, of whom 10 were excluded: eight because the biopsy specimen did not have the number of glomeruli described in the pathology report; one because the biopsy was of a renal nodule; and two because their records were not found in the registry of the Nephropathology Institute.

Demographic data, physical examination data, laboratory test results, and biopsy data (kidney biopsied, number of needle passes, quality of the material collected, number of patients discharged after the procedure, and number of patients referred to hospital emergency care) are listed in Table 1. Table 2 lists the indications for renal biopsy. Table 3 lists the pathology diagnoses, the total number of glomeruli obtained, and the percentage of sclerotic glomeruli.

**Table 1:** Demographic characteristics and characteristics of the material obtained from 234 patients undergoing ultrasound-guided percutaneous renal biopsy.

Characteristic	n (%) or range	Mean ± SD
Male	119 (50.85)	-
Female	115 (49.15)	-
Age (years)	8-83	48.01 ± 16.51
INR	1.0-1.3	1.04 ± 0.067
APTT (s)	20-35	27.4 ± 4.85
Platelets (n/mm^3^)	106,000-317,000	272,260 ± 208,089
Creatinine (mmol/L)	0.0345-0.50	0.248 ± 1.547
Urea (mmol/L)	3.16-37.96	8.82 ± 5.06
Systolic blood pressure (mmHg)	90-180	134.4 ± 17.47
Diastolic blood pressure (mmHg)	60-120	83.1 ± 8.97
Heart rate (bpm)	54-105	77.5 ± 9.3
Kidney punctured		
Native right	9 (4.70)	
Native left	216 (92.13)	
Transplanted	7 (2.09)	
Number of punctures	1-6	2.13 ± 0.74
1	41 (17.52)	
2	121 (51.71)	
3	57 (24.36)	
4	9 (3.85)	
5	2 (0.85)	
6	1 (0.43)	
Time to discharge (hours)	1-5	2.43 ± 0.93
Referral to emergency care		
Yes	2 (0.85)	
No	232 (99.15)	
Material considered sufficient		
Yes	224 (95.73)	
Partia Illy	8 (3.42)	
No (non-representative)	2 (0.85)	

SD, satandard deviation.

**Table 2:** Indications for ultrasound-guided renal biopsy in 234 consecutive patients with renal parenchymal disease.

Clinical indication	n (%)
Proteinuria (< 3 g/24 h or unspecified)	82 (28.0)
Worsening renal function	60 (20.5)
Hematuria of glomerular origin	54 (18.4)
Nephrotic proteinuria (> 3 g/24 h)	41 (14.0)
ANA+, SLE, or suspected autoimmune disease	33 (11.3)
Unspecified glomerulonephritis	12 (4.1)
Hypertension	9 (3.1)
Diabetes mellitus	4 (1.4)
Monoclonal gammopathy	1 (0.3)
Suspected FSGS	1 (0.3)
Renal graft dysfunction	1 (0.3)
Total	293 (100)

ANA, antinuclear antibody; SLE, systemic lupus erythematosus; FSGS, focal segmental glomerulosclerosis.

Note: The total is greater than 234 because several requests listed more than one indication.

**Table 3:** Main diagnoses and number of glomeruli obtained (as reported in the pathology report, including light microscopy and immunofluorescence) in 234 ultrasound-guided renal biopsies performed for clarification of parenchymal disease.

Main histopathological diagnosis	n (%)	Total number of glomeruli Mean (range)	Number of sclerotic glomeruli Mean (%)
IgA nephropathy	53 (22.46)	36(11-100)	8 (22.6)
Minimal change disease	42 (17.95)	40 (9-90)	3 (9.1)
Chronic tubulointerstitial nephritis	30 (12.82)	34 (6-97)	13 (35.9)
Membranous glomerulopathy	20 (8.55)	36 (1-86)	6 (11.5)
Diabetic nephropathy	20 (8.55)	40 (14-75)	19 (44.6)
Focal segmental glomerulosclerosis	19 (8.12)	35 ( 21-80)	14.7 (34.4)
Lupus nephropathy (all classes)	18 (7.69)	38 (13-68)	6.2 (15.5)
Membranoproliferative glomerulonephritis	13 (5.59)	51(14-186)	20 (21.6)
Amyloidosis	4 (1.71)	51 (30-76)	1.5 (1.47)
Inconclusive	4 (1.71)	49 (31-61)	4 (11.9)
Crescentic glomerulonephritis (any type)	3 (1.28)	48 (40-52)	20.7 (41.6)
Glomerulopathy with dominant C3	2 (0.85)	27 (27-28)	13 (47.1)
Hypertensive vasculopathy (poorly representative sample)	2 (0.85)	19 (4-33)	8.5 (47.7)
Rapidly progressive glomerulonephritis (all types)^*^	1 (0.43)	48	20 (41.7)
Nodular glomerulosclerosis due to monoclonal gammopathy with kappa light chain deposition^*^	1 (0.43)	28	7(25)
Normal (renal dysfunction in the context of Erdheim-Chester disease)^*^	1 (0.43)	7	0(0)
Subacute/chronic thrombotic microangiopathy^*^	1 (0.43)	53	6 (11.3)
Total or mean	234 (100)	38(1-186)	9.7 (23.0)

* Range not shown because there was only one patient with this diagnosis.

The material was considered sufficient for a definitive diagnosis in 230 (98.3%) of the 234 renal biopsies analyzed. The four inconclusive cases were due to negativity on the immunofluorescence evaluation. In three of those cases, no relevant changes were observed under light microscopy. In one of the four cases, sclerosis was observed in 42% of the glomeruli and there were retractions of glomerular tufts, together with dilation of Bowman’s capsule and atrophy of the tubular epithelium with discrete interstitial fibrosis, findings that are considered nonspecific.

In two (0.85%) of the 234 cases evaluated, symptomatic bleeding occurred at two hours after the biopsy, and those two patients were transferred to the hospital emergency room. One of those patients was a 43-year-old woman with a perirenal hematoma in whom the indication for biopsy was suspected lupus nephritis and for whom the following laboratory test results were obtained: INR, 1.08; APTT, patient 33 s/control 33 s; platelets, 215,000 mm^3^; serum urea, 88 mg/dL (14.65 mmol/L); serum creatinine, 3.08 mg/dL (0.273 mmol/L); blood pressure on admission, 140 × 90 mmHg; and heart rate, 92 bpm. In that patient, the left kidney was punctured, two fragments were collected, and 15 glomeruli were obtained, nine of which were sclerotic (an adequate sample). The histopathologic diagnosis was immunoglobulin A (IgA) nephropathy (Berger’s disease). The patient was transferred to the hospital emergency room, and a computed tomography (CT) scan of the abdomen performed at four hours after admission revealed a 10 cm^3^ increase in the volume of the perirenal hematoma. A blood transfusion was necessary because of a low red blood cell count (a pre-examination blood count indicated mild anemia). No surgical intervention was required, and the patient remained hemodynamically stable throughout the hospital stay. Another CT scan, performed the following day, showed a slight increase in the volume of the perirenal hematoma (to approximately 10 mL). The patient was discharged on post-admission day three, after an uneventful evolution and another CT scan showing that her condition was stable in relation to the previous examination. The other patient was a 34-year-old male in whom the indication for biopsy was type I diabetes mellitus with progressive worsening of renal function and nephrotic syndrome. The laboratory test results were as follows: INR, 1.0; APTT, patient 28 s/control 33 s; platelets, 228,000 mm^3^; serum urea, 109.0 mg/dL (18.15 mmol/L); serum creatinine, 3.03 mg/dL (0.268 mmol/L); proteinuria, 13.9 g in 24 h; blood pressure, 150 × 90 mmHg; and heart rate, 73 bpm). The left kidney was punctured, two fragments were collected, and 43 glomeruli were obtained, of which 16 were sclerotic (an adequate sample). The histopathology findings were consistent with diabetic nephropathy. The patient complained of severe pain 15 min after the procedure. Another ultrasound evaluation revealed a perirenal collection with an estimated volume of 80 cm^3^, with no signs of significant blood reflux on Doppler. Six ampoules of desmopressin were administered, as well as 1 g of intravenous dipyrone, without substantial improvement. One 50-mg ampoule of tramadol hydrochloride was administered, but the patient continued to complain of severe pain. The diagnostic hypothesis was renal colic due to distension of the capsule by a hematoma. Yet another ultrasound evaluation, performed 90 min after the biopsy, showed stability of the collection, which had an estimated volume of 80 cm^3^. Although hemodynamically stable, the patient complained of severe pain and was therefore transferred to the hospital emergency room, where a CT scan was performed and showed a controlled hematoma. The patient was medicated and was discharged, pain-free, in the evening of the same day.

## DISCUSSION

Renal biopsy plays a crucial role in the diagnosis of renal parenchymal diseases, being considered the gold standard for the identification, staging, and prognosis of such diseases. Renal biopsy is particularly valuable in cases of nephrotic-range proteinuria, as well as in patients with subnephrotic proteinuria, who are at substantial risk of progression to stage 5 chronic kidney disease and death, making diagnostic accuracy essential for guiding individualized treatment^**(^11^,^12^)**^. In the context of systemic diseases, such as systemic lupus erythematosus, renal biopsy is essential to confirm the diagnosis of lupus nephritis and to assess features of activity and chronicity that inform decisions regarding treatment and prognosis^**(^13^)**^. Renal biopsy is also essential in patients with type 2 diabetes mellitus, helping differentiate between diabetic nephropathy and other nondiabetic renal diseases that can require specific treatments^**(^14^)**^. Renal biopsy is also helpful in the classification of glomerular diseases, such as IgA nephropathy and antineutrophil cytoplasmic antibody-associated vasculitis, allowing prediction of chronic kidney disease progression and response to therapy. Therefore, renal biopsy is an essential diagnostic tool in nephrology practice, providing critical information that can significantly alter the clinical management and prognosis of renal parenchymal diseases^**(^11^,^15^,^16^)**^.

Counting the number of glomeruli obtained in a renal biopsy is essential for assessing the quality of the material obtained and for increasing the reliability of the diagnosis of primary and secondary parenchymal nephropathies. Hematuria with target cells (dysmorphic red blood cells) and most cases of proteinuria both result from glomerular injury. For a renal biopsy specimen to have value, the ideal is that 10 glomeruli are analyzed by hematoxylin-eosin staining under light microscopy and 10 more are analyzed by immunofluorescence in order to detect the deposition of immunoglobulin and complement. The minimum number of glomeruli required increases in nephrotic syndromes, which require at least 20 nonsclerotic glomeruli for an adequate analysis. The glomeruli can be counted under conventional light microscopy in fresh specimens immediately after collection, being seen as small, rounded formations that are reddish (when containing red blood cells) or whitish (when filled with proteins or other substances). Although this evaluation occasionally fails, it is the most widely used technique.

In the material obtained in our study sample, we observed a relatively high predominance of sclerotic glomeruli. We attribute that not only to the possibility that the nephrologists felt more confident in indicating ultrasound-guided renal biopsy in cases that were more complex or advanced (given the low incidence of significant adverse events historically reported by our team) but also to the difficulty in accessing care by specialists/nephrologists due to socioeconomic or geographic factors.

Since the 1970s, ultrasound has been used in order to guide biopsies, resulting in greater safety and the acquisition of better specimens for analysis. At our facility, we have been performing ultrasound-guided biopsies since 1988^**(^3^)**^. It has been demonstrated that such experience has a beneficial effect on the quality of the material obtained^**(^2^)**^.

Since 2013, the same pathologist has been monitoring the ultrasound-guided renal biopsies performed by the radiology team at our facility, examining the fresh specimens obtained immediately after the biopsy, counting the glomeruli and providing guidance on whether or not more material was needed. During this process, we gradually understood that the material with the most glomeruli was obtained when the needle was directed to the subcapsular region immediately below the renal surface, which corresponds to the renal cortex. In the literature, this method is referred to as the cortical tangential approach^(4-8,17)^, which has been progressively adopted at various centers because it has proven more effective than the conventional procedure for obtaining adequate renal samples. Using that approach in biopsies of transplanted kidneys, Patel et al.^(8)^ obtained a mean of 21.7 glomeruli, comparable to the 28.6 obtained in our study. We also observed that 16-gauge tru-cut needles are the ones that result in better specimens and a high degree of safety^**(^18^)**^. Specimens obtained by puncture with an 18-gauge needle are thinner, with fewer glomeruli, increasing the number of passes required to obtain sufficient material. Because 14-gauge needles are thicker, their use increases the risk of bleeding caused by inadvertent injury to the arcuate or interlobar artery.

The use of real-time ultrasound imaging, together with color Doppler, is essential to improve the safety and efficacy of biopsy, provided that the examiner has experience with the method^**(^19^)**^. However, the use of ultrasound guidance alone is not sufficient to completely avoid adverse hemorrhagic events if the needle is not directed to the renal cortex. If the needle is triggered in the most central portion of the kidney, there is a risk of injuring interlobar or segmental arteries, substantially increasing the risk of severe hemorrhage, which reportedly occurs in up to 40% of cases, especially when CT guidance is used^(20,21)^.

Notably, the incidence of significant adverse events was low in our study sample, because of the high quality of the material obtained by ultrasound-guided biopsy. As previously stated, considerable bleeding occurred in only two patients. In one of those patients, the bleeding was classified as mild acute and managed conservatively. In the other patient, it was classified as moderate acute and managed with blood transfusion^**(^22^)**^, without the need for radiological or surgical intervention. There were no late adverse events within the first week after biopsy, which is important when the biopsy is performed in an outpatient setting. The frequency of adverse events in our study sample is lower than that found in a meta-analysis published in 2020^**(^23^)**^, which analyzed 87 articles describing a collective total of 118,064 image-guided renal parenchymal biopsies. That meta-analysis found that hematomas occurred in 11% of cases, local pain occurred in 4.3%, bleeding requiring transfusion occurred in 1.6%, bleeding requiring radiological or surgical intervention occurred in 0.3%, and death occurred in 0.06%. The authors observed that significant adverse events were most common among hospitalized patients and those treated in the context of a medical emergency. Although some patients in our sample were hospitalized and had to go to the clinic for the procedure, the biopsy was elective in most cases. Patients with elevated serum creatinine-typically greater than 5 mg/dL (0.442 mmol/L)-or elevated serum urea greater than 100 mg/dL (16.65 mmol/L)-constitute another group of greater concern because of the hemorrhagic risk associated with platelet dysfunction. However, in our sample, we did not find the frequency of adverse events to be higher in such patients.

Our study has limitations, including the lack of a control group of patients undergoing renal biopsy with a perpendicular approach, a method we employed before migrating to the tangential approach. However, at that time we used 18-gauge needles to minimize the risk of hemorrhage. In the tangential approach, the needle is inserted only into the cortex, in a region without large vessels (identifiable on Doppler ultrasound), which allows us to safely use the larger 16-gauge needles. However, that makes it difficult to compare the two approaches. Therefore, we chose to report only the results of the tangential approach in this study, recognizing that a clear demonstration of the superiority of one technique over the other would require a prospective randomized study and that the extremely low complication rate of the tangential approach would make such a study ethically controversial. Most articles using the conventional approach to renal biopsy suggest immediate monitoring of patients (for ≤ 4 h) after the procedure, given that adverse events rarely occur > 24 h after renal biopsy. We did not encounter any complications within one week after the biopsy, nor did the patients or referring physicians report any adverse events occurring thereafter. However, we did not monitor for such events, which could be considered a limitation of our study. Another limitation of our study is that the population studied consisted of patients undergoing renal biopsy as an elective procedure at a private clinic. Although some were referred via the Brazilian Unified Health Care System, most had health insurance or were able to pay for the biopsy and the pathology study. However, some of the authors worked in a public hospital, where they performed renal biopsy using the same approach, with the difference that there was not always a pathologist in the room during the puncture, which is why we excluded such procedures from our analysis, to make it more homogeneous. Another possible selection bias is that we excluded patients with coagulation disorders, unless the disorder had been corrected. However, that practice was applied in all of the comparable articles we read. Because biopsy is generally an elective procedure, it is possible and desirable to correct any bleeding disorder before the procedure. However, we did not analyze that subgroup separately, which could be done in a prospective study. Finally, to validate our results, we suggest a prospective multicenter study involving different populations and physicians, following the same examination protocol, including reporting of and monitoring for adverse events.

In conclusion, when indicated and performed appropriately, percutaneous renal biopsy is a safe and effective method for diagnosing parenchymal nephropathies and systemic diseases with renal involvement. Given its safety and the excellent specimens obtained, the cortical tangential approach should be the technique of choice, even in the presence of renal dysfunction and significant glomerular sclerosis.
